# Elevated antiphospholipid antibody titers and adverse pregnancy outcomes: analysis of a population-based hospital dataset

**DOI:** 10.1186/1471-2393-9-11

**Published:** 2009-03-16

**Authors:** James Nodler, Surjit R Moolamalla, Elizabeth M Ledger, Bahij S Nuwayhid, Zuber D Mulla

**Affiliations:** 1 Department of Obstetrics and Gynecology, The University of Alabama at Birmingham, Birmingham, Alabama, USA; 2 Department of Obstetrics and Gynecology, Paul L. Foster School of Medicine, Texas Tech University Health Sciences Center, El Paso, Texas, USA; 3 Department of Epidemiology and Biostatistics, University of South Florida College of Public Health, Tampa, Florida, USA

## Abstract

**Background:**

The primary objective of this study was to determine if elevated antiphospholipid antibody titers were correlated with the presence of preeclampsia/eclampsia, systemic lupus erythematosus (SLE), placental insufficiency, and a prolonged length of stay (PLOS), in women who delivered throughout Florida, USA.

**Methods:**

Cross-sectional analyses were conducted using a statewide hospital database. Prevalence odds ratios (OR) were calculated to quantify the association between elevated antiphospholipid antibody titers and four outcomes in 141,286 women who delivered in Florida in 2001. The possibility that the relationship between elevated antiphospholipid antibody titers and the outcomes of preeclampsia/eclampsia, placental insufficiency, and PLOS, may have been modified by the presence of SLE was evaluated in a multiple logistic regression model by creating a composite interaction term.

**Results:**

Women with elevated antiphospholipid antibody titers (n = 88) were older, more likely to be of white race and not on Medicaid than women who did not have elevated antiphospholipid antibody titers. Women who had elevated antiphospholipid antibody titers had an increased adjusted odds ratio for preeclampsia and eclampsia, (OR = 2.93 p = 0.0015), SLE (OR = 61.24 p < 0.0001), placental insufficiency (OR = 4.58 p = 0.0003), and PLOS (OR = 3.93 p < 0.0001). Patients who had both an elevated antiphospholipid antibody titer and SLE were significantly more likely than the comparison group (women without an elevated titer who did not have SLE) to have the outcomes of preeclampsia, placental insufficiency and PLOS.

**Conclusion:**

This exploratory epidemiologic investigation found moderate to very strong associations between elevated antiphospholipid antibody titers and four important outcomes in a large sample of women.

## Background

The antiphospholipid syndrome (APS) is described as an autoimmune disorder defined by both clinical and laboratory criteria. Clinical criteria include vascular thrombosis as well as unexplained fetal death, preeclampsia, and eclampsia [1]. Laboratory criteria include the presence of medium to high titers of lupus anticoagulant, anticardiolipin, or anti-β_2 _glycoprotein-I antibodies [1]. APS is now thought to be a systemic disease, affecting multiple organs and systems [2]. Multiple medical and obstetric complications are commonly associated with APS such as preeclampsia, eclampsia, placental insufficiency, thrombocytopenia, stroke, transient ischemic attack, pulmonary embolism, livedo reticularis, Libman-Sacks endocarditis, multi-infarct dementia, migraine headache, transverse myelitis, cutaneous ulcers, venous thrombosis, and deep-vein thrombosis as well as other maladies [2-5].

Systemic lupus erythematosus (SLE) has historically been strongly linked with APS. APS was first described as being a subset of SLE [3]. Patients that have APS and SLE are termed "secondary APS," while those that have APS without clinical overt SLE or any symptom of SLE are termed "primary APS" [4]. The prevalence of IgG anticardiolipin antibodies in SLE patients has been shown to be as high as 22.8%, while the prevalence of IgM and IgG anti-β_2 _glycoprotein-I antibodies in SLE patients has been shown to be as high as 20% [4]. Many studies have examined whether having APS with coexisting SLE causes a greater increase in adverse outcomes such as pregnancy loss than having APS alone [3]. Studies have shown that having SLE and APS puts one at higher risk for thrombosis than having either SLE or APS alone [3].

It is well known that APS and SLE increase maternal and perinatal morbidity [6,7]. What is not known is the demographic and epidemiologic profile of patients with increased antiphospholipid (AP) antibody titers, and the prevalence of co-morbidities associated with the increased titers. Also, certain populations may be at increased risk for elevated AP antibody titers and might benefit from more advanced diagnostic and therapeutic interventions.

We conducted an epidemiologic study to determine if elevated antiphospholipid antibody titers (a criterion for diagnosis of APS) are correlated with the presence of preeclampsia and eclampsia, SLE, placental insufficiency, and a prolonged length of stay (PLOS). The setting of the analysis was a statewide hospital database. To our knowledge this is the first investigation of its kind using inpatient data from the Florida Agency for Health Care Administration.

## Methods

### Source of patients/Inclusion criteria

Retrospective analyses were performed using a hospital discharge dataset that was obtained from the Florida Agency for Health Care Administration (Tallahassee, Florida). This public-use database includes discharge summaries from all non-federal Florida hospitals except state tuberculosis and state mental health hospitals. After data are entered into this system, they are subjected to formatting and logic checks. The primary hospital submitting patient information must then certify the data are correct and verify the accuracy of a summary report before it is released by the Agency for Health Care Administration.

This dataset contained clinical and demographic information for 2,343,330 patients who were hospitalized for at least one day and discharged in calendar year 2001. The principal diagnosis and up to nine secondary diagnoses were coded using the *International Classification of Diseases, Ninth Revision, Clinical Modification *(ICD-9-CM). Up to and including ten procedures (a principal procedure field plus nine secondary procedures) could have been recorded. Procedures were coded using the ICD-9-CM.

The current study focused on women who delivered during their hospital stay. Women who had one of the following ICD-9-CM procedure codes in any of their ten procedure fields were considered to have delivered a child during that admission: 72, 72.1, 72.21, 72.29, 72.31, 72.39, 72.4, 72.51, 72.52, 72.53, 72.6, 72.71, 72.79, 72.8, 72.9, 72.9, 73.3, 73.59, 74, 74.1, 74.2, 74.4, 74.99.

### Definitions of APS and outcomes

The ICD-9-CM code for antiphospholipid antibody syndrome is 795.79. The description of this code in the tabular list of ICD-9-CM is, "Other and unspecified nonspecific immunological findings: Raised antibody titer, Raised level of immunoglobulins."

Originally we aimed to study the following 14 outcomes in addition to the aforementioned four outcomes; however, there were either too few cases overall (< 5) and/or too few patients (< 5) who had both the exposure of elevated antiphospholipid antibody titers and the outcome of interest to conduct reliable multivariate regression analyses: stroke, transient ischemic attack, pulmonary embolism, arterial embolism and thrombosis, thrombocytopenia, autoimmune hemolytic anemia, livedo reticularis, Libman-Sacks endocarditis, multi-infarct dementia, migraine headache, transverse myelitis, cutaneous ulcers, venous thrombosis, and deep-vein thrombosis. Maternal records are not linked to their respective infant records in this database and therefore outcomes such as fetal death could not be evaluated.

If the patient's principal discharge diagnosis or any of the nine secondary diagnosis fields contained code 795.79 then the patient was considered to have elevated antiphospholipid antibody titers. Similarly, preeclampsia and eclampsia were defined as ICD-9-CM code 642.40, 642.41, 642.42, 642.43, 642.44, 642.50, 642.51, 642.52, 642.53, 642.54, 642.60, 642.61, 642.62, 642.63, 642.64, 642.70, 642.71, 642.72, 642.73, or 642.74. Patients were classified as having SLE if code 710.0 was found in any of the ten diagnosis slots. The presence of codes 656.50, 656.51, and 656.53 in the electronic record identified women with placental insufficiency. The 656.5 category is also described as "poor fetal growth," "light-for-dates," and "small-for-dates" in the tabular ICD-9-CM list.

PLOS was defined as a length of stay (recorded in days) that was greater than the 75^th ^percentile in our sample. A LOS of 3 days represented the 75^th ^percentile in our study. Dichotomizing LOS using the 75^th ^percentile has been used in previous clinical epidemiology analyses [8,9].

### Data analysis

A cross-sectional study was conducted to determine if the prevalence of the four outcomes stated above varied by elevated antiphospholipid antibody titer status. Data were analyzed using The SAS System for Windows 9.1.3 (The SAS Institute, Inc., Cary, North Carolina). We performed logistic regression using PROC LOGISTIC. Crude and adjusted prevalence odds ratios (OR) were calculated for preeclampsia/eclampsia, SLE, placental insufficiency, and PLOS. An OR was considered statistically significant if the 95% confidence interval for the population OR excluded the null value of one.

The following variables were evaluated for confounding for each of the four outcomes: Age (in years), race-ethnicity, and health insurance status. Race-ethnicity was originally an eight-level variable (7 racial ethnic groups plus a category for missing). This variable was converted to a four-level categorical variable: White Hispanic, Black non-Hispanic, Other racial/ethnic group, and White non-Hispanic (referent). Asian and Pacific Islanders, Black Hispanics, American Indians/Eskimo/Aleut, and patients of Other racial/ethnic groups were collapsed into a new Other group. Our dichotomous health insurance variable was created using the 14-level principal payer variable found in the database: Patients whose primary insurer was Medicaid or a Medicaid health maintenance organization were compared to other patients. The "other" group included various payers such as Medicare, charity, and commercial insurance.

When preeclampsia/eclampsia was the outcome, the following variables were also considered potential confounders: Obesity or morbid obesity (defined as the occurrence of ICD-9-CM codes 278.00 or 278.01 in any of the ten discharge diagnosis fields), diabetes, and primigravid status. Diabetes was defined as the presence of ICD-9-CM code 250.0 to 250.9 (type I and II diabetes) or ICD-9-CM code 648.8 (gestational diabetes) in any of the discharge diagnosis fields. The database did not contain detailed information on the patient's gravidity and parity; however, several ICD-9-CM codes were used to attempt to identify a primigravida, which was treated as a potential confounder of the association between elevated antiphospholipid antibody titers and preeclampsia. These codes were 659.5 (elderly primigravida – a woman who will be ≥ 35 years of age at the expected date of delivery), 659.8 (young primigravida – a woman who will be < 16 years of age at the expected date of delivery), V22.0 (supervision of a normal first pregnancy), V23.81 (supervision of a high-risk pregnancy, elderly primigravida), and V23.83 (supervision of a high-risk pregnancy, young primigravida). For the outcome of PLOS, diabetes and gestational diabetes (defined above) were considered potential confounders.

Backward elimination was utilized to identify confounders for inclusion in the four final models. The significance level for potential confounders to remain in the model was 0.20. Mickey and Greenland have shown using Monte Carlo simulations that this type of significance testing method can perform acceptably in selecting confounders if the significance level is set much higher than conventional levels (for example, 0.20 or greater) [10]. The method that we employed generally agrees with the change-in-estimate method, which is popular among epidemiologists, as long as the alpha is 0.20 or higher [10]. (The change-in-estimate method does not rely on p-values but rather the change in the OR associated with the exposure of interest as one or more variables are added to the model) [11].

In the final phase of the statistical analysis a composite interaction variable was created to determine if there was an interaction between elevated antiphospholipid antibody titers and SLE when the following variables were the outcomes: preeclampsia/eclampsia, placental insufficiency, and PLOS [12]. To clarify, the following four risk groups were formed to evaluate this potential interaction: Had elevated AP antibody titers and SLE, had elevated AP antibody titers but not SLE, no elevated AP antibody titers but had SLE, and did not have elevated AP antibody titers and did not have SLE. The last group served as the referent or comparison group. The assessment of confounding was identical to the procedure described above.

It is possible that several of our independent variables were highly correlated (such as obesity and diabetes), a phenomenon known as collinearity. Collinearity can result in inaccurate estimates of regression coefficients and p-values and can lead to reduced statistical power [13,14]. Our analysis of the tolerances did not detect any collinearity [15].

A total of 143,970 women who delivered during 2001 in Florida were captured by this database. After deleting the records of 2632 women who had missing values for race-ethnicity, there were 141,338 records remaining. In an attempt to only include one record per patient a small number of women (n = 52) who were transferred to a short-term general hospital or intermediate care facility were deleted. This strategy was utilized because the dataset lacked a unique patient identifier. The final sample size was 141,286. Analysis of this dataset was approved by the Institutional Review Board of the Texas Tech University Health Sciences Center, El Paso, Texas.

## Results

Selected demographic and clinical characteristics of the sample are shown in Table 1. Women who carried the diagnosis of elevated AP antibody titers comprised 0.06% of the study sample (88/141,286). These women were older and more likely to be of white race than women who did not have elevated AP antibody titers (Table 1). According to the principal payer code in the database, elevated AP antibody titer patients were less likely to be Medicaid beneficiaries than patients free of elevated AP antibody titers. The crude prevalence rates of all four outcomes were higher in the elevated AP antibody titer group. Approximately 0.8% and 0.2% of the entire sample had an ICD-9-CM code indicating obesity and primigravida status, respectively (data not shown).

**Table 1:** Demographic and Clinical Characteristics of 141,286 Women who Delivered throughout Florida in 2001

	**Elevated Antiphospholipid Antibody Titers**	
	Present (n = 88)	Absent (n = 141,198)

Variable	Number (Percent)	Number (Percent)

Age (years) *		

11–19	2 (2.27)	16,155 (11.44)

20–29	33 (37.50)	72,316 (51.22)

30–39	47 (53.41)	48,751 (34.53)

40–49	6 (6.82)	3,964 (2.81)

≥ 50	0 (0.0)	12 (0.01)

		

Race-ethnicity		

Black	12 (13.64)	32,940 (23.33)

White Hispanic	15 (17.05)	29,520 (20.91)

White Non-Hispanic	57 (64.77)	70,529 (49.95)

Other	4 (4.55)	8,209 (5.81)

		

Medicaid		

Yes	16 (18.18)	58,333 (41.31)

No	72 (81.82)	82,865 (58.69)

		

Hospital mortality	0 (0.0)	21 (0.01)

		

Preeclampsia or eclampsia	10 (11.36)	6,265 (4.44)

		

Systemic lupus erythematosus	5 (5.68)	126 (0.09)

		

Placental insufficiency	6 (6.82)	2,347 (1.66)

		

Prolonged length of stay (> 3 days)	34 (38.64)	18,808 (13.32)

* Median age (range) of APS patients = 30.5 years (18 years-43 years) and median age of patients free of APS (range) = 27 years (11 years-54 years)

Overall 6275 women carried the diagnosis of preeclampsia/eclampsia (Table 1), and according to the ICD-9-CM nomenclature, these women were classified into four groups: 179 had eclampsia (2.85%), 610 had preeclampsia or eclampsia superimposed on pre-existing hypertension (9.72%), 2052 had severe preeclampsia (32.70%), and 3434 had mild or unspecified preeclampsia (54.73%) (data not shown).

Crude (unadjusted) and adjusted prevalence ORs are reported in Table 2. The ORs quantified the association between elevated AP antibody titers and four outcomes: preeclampsia or eclampsia, SLE, placental insufficiency, and PLOS. Each of the four adjusted ORs was statistically significant indicating that random chance was not a likely explanation of the result. The adjusted OR for preeclampsia/eclampsia revealed a moderate correlation with elevated AP antibody titers while the ORs for placental insufficiency and PLOS (both > 3.9) suggested strong relationships between elevated AP antibody titers and these two outcomes. The strongest adjusted OR in Table 2 is for the outcome of SLE: 61.24 (95% confidence interval: 24.29–154.44). The latter OR was adjusted for age and race-ethnicity.

**Table 2:** Association between Elevated Antiphospholipid Antibody Titers (Present vs. Absent) and Four Adverse Outcomes in 141,286 Women Delivering in Florida in 2001

	Unadjusted		Adjusted	
Outcome	Odds Ratio [95% CI*]	P-value	Odds Ratio [95% CI^a^*]	P-value

**Preeclampsia or Eclampsia**	2.77 [1.44, 5.35]	0.002	2.93^† ^ [1.51, 5.69]	0.0015

				

**Systemic lupus erythematosus**	67.45 [26.90, 169.12]	< 0.0001	61.24^‡^ [24.29, 154.44]	< 0.0001

				

**Placental insufficiency**	4.33 [1.89, 9.93]	0.0005	4.58^§^ [2.00, 10.51]	0.0003

				

**Prolonged length of stay**^¶^	4.10 [2.67, 6.30]	< 0.0001	3.93^∥^ [2.55, 6.06]	< 0.0001

* CI: Confidence interval

^† ^Adjusted for race, age, Medicaid status, diabetes, obesity, and primigravida status

^‡ ^Adjusted for race and age

^§ ^Adjusted for race, age, and Medicaid status

^∥ ^Adjusted for race, age, Medicaid status, and diabetes

^¶ ^Defined as a length of stay of > 3 days

The adjusted ORs in Table 3 compare women in three risk groups (elevated AP antibody titers and SLE, elevated AP antibody titers and no SLE, no elevated AP antibody titers and SLE) to women who are free of both conditions. The results are read vertically for each outcome. Only five women had both elevated AP antibody titers and SLE noted in their discharge records (Table 3); however, having both conditions was a strong correlate of the following outcomes: preeclampsia/eclampsia, placental insufficiency, and PLOS.

**Table 3:** Adjusted Prevalence Odds Ratios for Three Outcomes: Evaluation of a Potential Interaction between Elevated Antiphospholipid Antibody Titers (EAAT) and Systemic Lupus Erythematosus (SLE)

	**Outcomes**					
	
	Preeclampsia or Eclampsia		Placental Insufficiency		Prolonged length of stay	
**Risk Factor Group**	Odds Ratio* [95% CI^†^]	P-value	Odds Ratio^‡^ [95% CI^†^]	P-value	Odds Ratio^§ ^ [95% CI^†^]	P-value

Has EAAT, and Has SLE (*n = 5*)	33.14 [5.29, 207.61]	0.0002	14.12 [1.58, 126.51]	0.018	9.68 [1.57, 59.87]	0.015

Has EAAT, No SLE (*n = 83*)	2.12 [0.98, 4.63]	0.058	4.06 [1.64, 10.03]	0.0025	3.72 [2.38, 5.83]	< 0.0001

No EAAT, Has SLE (*n = 126*)	3.29 [1.97, 5.52]	< 0.0001	4.61 [2.34, 9.09]	< 0.0001	3.69 [2.56, 5.31]	< 0.0001

No EAAT, and No SLE (*n = 141,072*)	1 [Reference]	--	1 [Reference]	--	1 [Reference]	--

* Adjusted for race, age, Medicaid status, diabetes, obesity, and primigravida status

^† ^CI = Confidence interval

^‡ ^Adjusted for race, age, and Medicaid status

^§ ^Adjusted for race, age, Medicaid status, and diabetes

## Discussion

Large hospital datasets allow investigators to conduct rapid exploratory studies that are population-based. Our statewide cross-sectional study revealed moderate to very strong associations between the risk factor of elevated AP antibody titers and four important outcomes in a large sample of women who delivered throughout Florida in calendar year 2001 (n = 141,286). Women who had elevated AP antibody titers had more than 60 times the odds of having SLE than women who did not have elevated AP antibody titers: Adjusted prevalence OR = 61.24 (p < 0.0001). This robust relationship is not unexpected. APS and SLE are both autoimmune diseases and the former was initially described as a subset of the latter [3]. While both are characterized by inflammation, APS is typified more by hypercoagulability and thrombotic events [3].

A strong correlation between elevated AP antibody titer status and PLOS was also found (crude OR = 4.10). This association persisted even after adjusting for age, race, Medicaid status, and the presence of diabetes (adjusted OR = 3.93). Length of stay is a recognized measure of disease severity when evaluating pregnancy-related morbidity [16].

A relationship between APS and an increased risk of developing preeclampsia is known; however, whether or not there is an association between high titers of AP antibodies and preeclampsia in the absence of APS is unclear [5]. To clarify, women who satisfy both the clinical and laboratory criteria for APS have a higher incidence of preeclampsia, but it is uncertain if merely a raised titer of AP antibodies is linked to preeclampsia [5]. We report an unadjusted prevalence of preeclampsia of 11.4% in women with a raised antibody titer of AP antibodies and a prevalence of preeclampsia of 4.4% in women who do not have a raised titer (Table 1). Clark *et al*. recently summarized the literature on the possible association between high titers of circulating AP antibodies and preeclampsia [5]. They report that the incidence of preeclampsia in women with positive tests for AP antibodies ranges from 11% to 50%. In contrast, the incidence of preeclampsia in women who test negative was 2% to 4%.

A possible reason for the conflicting data arising from studies of AP antibodies in preeclamptic women is differing thresholds for a positive test for AP antibodies and a lack of standardization of the assays used in these investigations [5]. According to Clark and colleagues, there is no agreement regarding what tests are best suited for screening for APS in both the general and pregnant population [5].

A strength of our investigation was the use of a large, statewide database obtained from the Florida Agency for Health Care Administration. The majority of civilian hospitals in Florida are required to report their discharge data to the Agency for Health Care Administration. To our knowledge this is the first study of its kind to report on Florida inpatients. This large data base includes 41,000 deliveries, so it is ideal for studying the epidemiology of rare diseases/events that might take several years to collect from prospective studies or case reports. Studies of hospitalized patients in which both the potential risk factor and the outcome are diseases are prone to Berkson's bias [17]. Berkson was a physician at the Mayo Clinic who reported the following phenomenon in 1946: Two diseases may appear to be correlated with one another in a hospital-based case-control study when in fact they are not simply because individuals with two or more conditions are more likely to be hospitalized than patients with one disease [17,18]. This selection bias may result in ORs that are biased upwards.

A limitation of this and most other cross-sectional studies is the doubt regarding whether or not temporality is intact: Did the suspected risk factor truly occur before the outcome or is it possible that the two are reversed? For example, it is possible that a patient may have been diagnosed with SLE some time before the onset of elevated AP antibody titers. A prospective study design would ensure that reverse causality bias was not in effect.

Another limitation of the current study is the lack of data on the accuracy of the ICD-9-CM coding in our database. Lydon-Rochelle et al. studied the accuracy of the reporting of particular maternal in-hospital diagnoses and intrapartum procedures in the state of Washington, USA [19]; however, the sensitivity and specificity of the coding of the conditions that we studied in this particular dataset are unknown. We did not have access to medical records to verify diagnoses. The prevalence of obesity and the proportion of our sample that was classified as primigravidas were much lower than expected. These codes may be underutilized. In addition, there are no ICD-9-CM codes identifying primigravidas between the ages of 16 and 34 years. In light of this situation residual confounding by both of these factors may be present.

A further limitation is that we do not know which laboratory tests were done, the anticardiolipin, lupus anticoagulant, the anti-B_2 _– GPI or another antiphospholipid test. The lupus anticoagulant test is difficult to perform and many times is not included. Additionally, we do not know the titers for these positive tests. When women with low titers are included, the outcome improves significantly. Because of this, many studies have suggested that only pregnant women with moderately or significantly elevated titers should be included.

We did conduct sensitivity analyses of misclassification of the exposure status (elevated antiphospholipid antibody titer status) using standard formulae [20]. For three of our four outcomes (preeclampsia, placental insufficiency, and PLOS) the corrected ORs were similar to those found in Table 2 (data not shown). For the outcome of SLE, the corrected ORs ranged from 12.75 to 15.60, values that were dramatically less than those found in Table 2 (ORs of 61.24 and 67.45). Nonetheless, the ORs for SLE corrected for various degrees of misclassification still indicate the presence of a very strong relationship.

Another drawback to this data base is that maternal and neonatal data are not linked, so we do not have data on IUGR or small for gestational age neonates. Also, this data base does not include the details of treatment modalities such as aspirin, heparin or corticosteroids and their effect on fetal and neonatal outcomes.

## Conclusion

Our epidemiologic study found that elevated AP antibody titers were positively associated with the presence of four major outcomes: preeclampsia/eclampsia, SLE, placental insufficiency, and PLOS. Attentive clinical care is required for best outcomes: surveillance to prevent placental insufficiency and to rule out intrauterine growth restriction and frequent clinical follow up to decrease morbidity associated with preeclampsia and eclampsia and intrauterine growth restriction. Antiphospholipid syndrome is one of the few treatable causes of pregnancy loss, and successful pregnancy rates of 70% or more can be achieved with appropriate treatment [21,22].

## Abbreviations

AP: Antiphospholipid; APS: Antiphospholipid syndrome; ICD-9-CM: International Classification of Diseases Ninth Revision Clinical Modification; OR: Odds ratio; PLOS: Prolonged length of stay; SLE: Systemic lupus erythematosus.

## Competing interests

The authors declare that they have no competing interests.

## Authors' contributions

JN and ZDM conceived the initial research questions. ZDM analyzed the data with input from the remaining authors. All authors participated in the interpretation of the results and the drafting and approval of the manuscript.

## Pre-publication history

The pre-publication history for this paper can be accessed here:


